# Identification of Antiviral Agents Targeting Hepatitis B Virus Promoter from Extracts of Indonesian Marine Organisms by a Novel Cell-Based Screening Assay

**DOI:** 10.3390/md13116759

**Published:** 2015-11-06

**Authors:** Atsuya Yamashita, Yuusuke Fujimoto, Mayumi Tamaki, Andi Setiawan, Tomohisa Tanaka, Kaori Okuyama-Dobashi, Hirotake Kasai, Koichi Watashi, Takaji Wakita, Masaaki Toyama, Masanori Baba, Nicole J. de Voogd, Shinya Maekawa, Nobuyuki Enomoto, Junichi Tanaka, Kohji Moriishi

**Affiliations:** 1 Department of Microbiology, Division of Medical Sciences, Graduate School of Interdisciplinary Research, University of Yamanashi, 1110 Shimokato, Chuo, Yamanashi 409-3898, Japan; E-Mails: atsuyay@yamanashi.ac.jp (A.Y.); yfujimoto@greiner-bio-one.co.jp (Y.F.); tomohisat@yamanashi.ac.jp (T.T.); kaorid@yamanashi.ac.jp (K.O.-D.); hirotake@yamanashi.ac.jp (H.K.); 2 Department of Chemistry, Biology and Marine Science, University of the Ryukyus, 1 Senbaru, Nishihara, Okinawa 903-0213, Japan; E-Mail: m.tamaki.4@jaist.ac.jp; 3 Department of Chemistry, Faculty of Science, Lampung University, Jl. Sumantri Brodjonegoro No. 1, Bandar Lampung 35145, Indonesia; E-Mail: andi.setiawan@fmipa.unila.ac.id; 4 Department of Virology II, National Institute of Infectious Diseases, 1-23-1 Toyama, Shinjuku-ku, Tokyo 162-8640, Japan; E-Mails: kwatashi@nih.go.jp (K.W.); wakita@nih.go.jp (T.W.); 5 Division of Antiviral Chemotherapy Center for Chronic Viral Disease, Graduate School of Medical and Dental Sciences, Kagoshima University, 8-35-1 Sakuragaoka, Kagoshima 890-8544, Japan; E-Mails: toyama@m2.kufm.kagoshima-u.ac.jp (M.T.); m-baba@m2.kufm.kagoshima-u.ac.jp (M.B.); 6 Naturalis, National Museum of Natural History, P.O. Box 9517, Leiden 2300 RA, The Netherlands; E-Mail: Nicole.devoogd@naturalis.nl; 7 The First Department of Internal Medicine, Faculty of Medicine, University of Yamanashi, 1110 Shimokato, Chuo, Yamanashi 409-3898, Japan; E-Mails: maekawa@yamanashi.ac.jp (S.M.); enomoto@yamanashi.ac.jp (N.E.)

**Keywords:** marine organism, hepatitis B virus, HBV, HBV core promoter, high-throughput screening, antiviral agent

## Abstract

The current treatments of chronic hepatitis B (CHB) face a limited choice of vaccine, antibody and antiviral agents. The development of additional antiviral agents is still needed for improvement of CHB therapy. In this study, we established a screening system in order to identify compounds inhibiting the core promoter activity of hepatitis B virus (HBV). We prepared 80 extracts of marine organisms from the coral reefs of Indonesia and screened them by using this system. Eventually, two extracts showed high inhibitory activity (>95%) and low cytotoxicity (66% to 77%). Solvent fractionation, column chromatography and NMR analysis revealed that 3,5-dibromo-2-(2,4-dibromophenoxy)-phenol (compound **1**) and 3,4,5-tribromo-2-(2,4-dibromophenoxy)-phenol (compound **2**), which are classified as polybrominated diphenyl ethers (PBDEs), were identified as anti-HBV agents in the extracts. Compounds **1** and **2** inhibited HBV core promoter activity as well as HBV production from HepG2.2.15.7 cells in a dose-dependent manner. The EC_50_ values of compounds **1** and **2** were 0.23 and 0.80 µM, respectively, while selectivity indexes of compound **1** and **2** were 18.2 and 12.8, respectively. These results suggest that our cell-based HBV core promoter assay system is useful to determine anti-HBV compounds, and that two PBDE compounds are expected to be candidates of lead compounds for the development of anti-HBV drugs.

## 1. Introduction

Hepatitis B virus (HBV) infection is a serious public health problem worldwide, with more than 240 million people estimated to be chronically infected [1]. Chronic infection with HBV leads to liver cirrhosis and hepatocellular carcinoma, which are adverse outcomes seen in untreated patients [2,3].

HBV is an enveloped DNA virus that belongs to the genus *Orthohepadnavirus* of the *Hepadnaviridae* family [4]. The infectious virion of HBV contains incompletely double-stranded and relaxed circular DNA (rcDNA), surrounded with a lipid bilayer and viral surface proteins. Following virus entry into hepatocytes, rcDNA migrates into the nucleus and is then converted into a covalently closed circular DNA (cccDNA), which encodes overlapping open reading frames (ORFs). The viral genes are transcribed under the control of four promoters (core, preS1, preS2/S, and X promoters) and two enhancer regions (enhancer I and enhancer II also referred as the core upstream regulatory sequence: CURS), and translated into the core protein (Hepatitis B core antigen: HBcAg), precore protein (Hepatitis B e antigen: HBeAg), surface proteins (Large S, Middle S and Small S protein), polymerase (reverse transcriptase and DNA-dependent DNA polymerase) and X protein. These viral regulatory elements play a role in transcriptions of 3.5, 2.4, 2.1 and 0.7 kb mRNAs. The mRNA with a size of 3.5 kb, which is termed pregenomic RNA (pgRNA), is packaged with the viral polymerase into a viral capsid consisting of core proteins. The pgRNA is enclosed with capsid proteins in cytoplasm and then reverse-transcribed into a negative-strand DNA in the cytoplasmic capsid. The transcription of pgRNA is regulated under the control of the core promoter, which consists of the basic core promoter and the upper regulatory region including negative regulatory region and CURS. Thus, the core promoter is responsible for HBV replication as well as the viral particle formation and is capable of being targeted for development of an effective HBV therapy [5,6,7,8,9,10].

The currently available antiviral agents for the treatment of chronic HBV infection are classified as follows: (1) immunomodulatory agents, such as conventional interferon-alpha and pegylated interferon-alpha; and (2) oral nucleoside/nucleotide analogues (NAs), such as three nucleoside (lamivudine, entecavir and telbivudine) and two nucleotide analogues (adefovir and tenofovir). Treatments with these agents are capable of preventing disease progression to liver cirrhosis and hepatocellular carcinoma, resulting in improvement of the survival rate of patients with chronic HBV infections [11,12,13]. However, interferon therapy is associated with major problems such as serious side effects, genotype-dependent treatment response and moderate antiviral activity, while long-term therapy using NAs promotes the emergence of drug-resistant viruses. In addition, the most serious problem is that currently available agents do not eradicate cccDNA, the template in transcription of HBV pgRNA and mRNA. Safer and more effective anti-HBV agents are still needed for efficient therapy [14,15].

Natural products including terrestrial plants and microbes have historically been sources for the development of various drugs targeting human diseases. Research on natural products has often included marine organisms because of the chemical and biological novelties of marine natural products. trabectedin (Yondelis^®^) and eribulin (Halaven^®^) are derived from chemical compounds isolated from marine organisms, and approved for anticancer therapy [16,17]. Ara-A (vidarabine) is a semisynthetic anti-herpes drug made from spongouridine isolated from the Caribbean sponge *Tethya crypta* [18,19].

In this study, we established a screening system to identify compounds inhibiting HBV core promoter activity and then screened 80 extracts of marine organisms collected from the coral reefs of Indonesia in order to identify anti-HBV agents.

## 2. Results and Discussion

### 2.1. Establishment of HBV Core Promoter Reporter Cell Line

**Figure 1:**
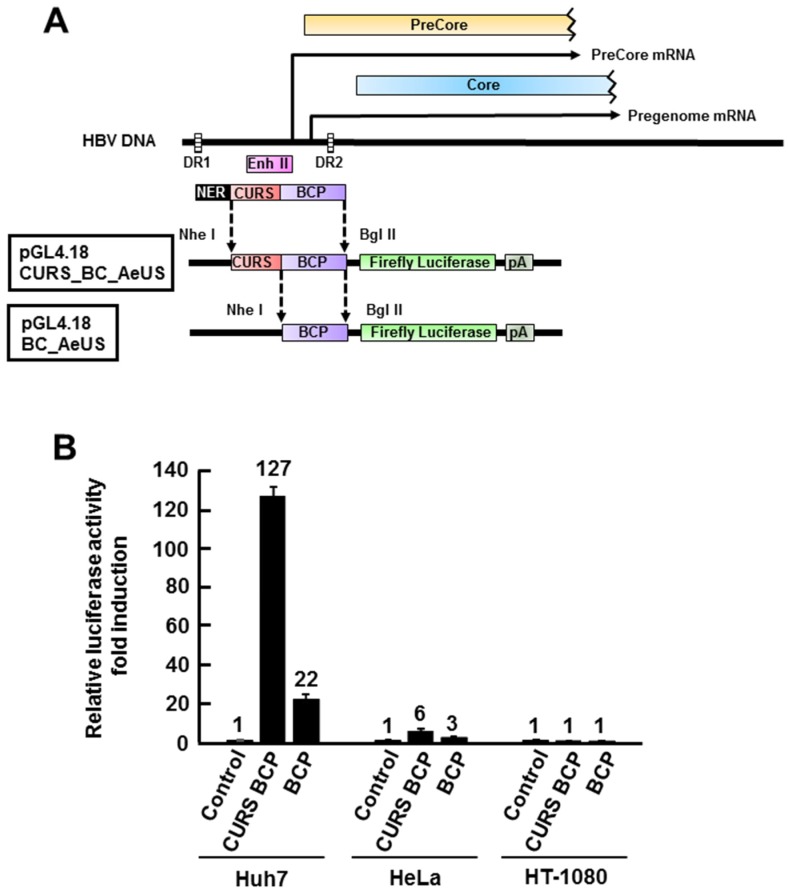
Development of Hepatitis B virus (HBV) core promoter reporter system. (**A**) Schematic representation of the firefly luciferase reporter plasmid pGL4.18 CURS_BC_AeUS and pGL4.18 BC_AeUS; (**B**) HBV core promoter activity in three cell lines. Each plasmid described above was transfected with phRG-TK into hepatic (Huh7) and non-hepatic (HeLa and HT-1080) cells. Luciferase activity was measured at 48 h post-transfection as described in the Experimental Section. Firefly luciferase activity was normalized with *Renilla* luciferase activity. Luciferase activity was expressed as a fold induction compared with the value of cells transfected with pGL4.18 [*luc2P*/Neo] empty control vector (control). The data shown in this panel are representative of three independent experiments. Error bars indicate standard deviation.

The core promoter consists of CURS and basal core promoter (BCP) (Figure 1A) and is responsible for transcription of 3.5 kb mRNA, pgRNA [4]. CURS negatively and positively regulates the promoter activity [4]. The region composed of both CURS and BCP or BCP only was cloned into pGL4.18 [*luc2P*/Neo] plasmid (Figure 1A). The resulting plasmids were designated as pGL4.18 CURS_BC_AeUS (CURS BCP) or pGL4.18 BC_AeUS (BCP) in this study (Figure 1A). The plasmid pGL4.18 CURS_BC_AeUS or pGL4.18 BC_AeUS was transfected with phRG-TK into human hepatoma cell line Huh7, human cervical cancer cell line HeLa, and human fibrosarcoma cell line HT-1080. The resulting cells were harvested 48 h post-transfection and suspended in lysis buffer in order to estimate luciferase activity. Previous findings suggested that HBV core promoter (CURS and BCP) is more active in hepatoma cell lines than other cell lines [6,20,21,22,23]. In this study, Huh7 cell line exhibited the highest luciferase activity under the control of CURS BCP or BCP among tested cell lines (Figure 1B). Moreover, the Huh7 cells transfected with pGL4.18 CURS_BC_AeUS exhibited 5-time higher luciferase activity than the cells transfected with pGL4.18 BC_AeUS (Figure 1B). These results suggest its potential for establishment of a cell-based screening assay based on HBV promoter activity. The plasmid pGL4.18 CURS_BC_AeUS was introduced into Huh7 cells again for establishment of a stable cell line. The transfected cells were incubated in the presence of G418 until colony formation. The Huh7 cell line exhibiting the highest luciferase activity was selected by colony isolation, and designated as Huh7 GL4.18 CURS_BC_AeUS.

### 2.2. Validation of Cell-Based HBV Core Promoter Assay

**Figure 2:**
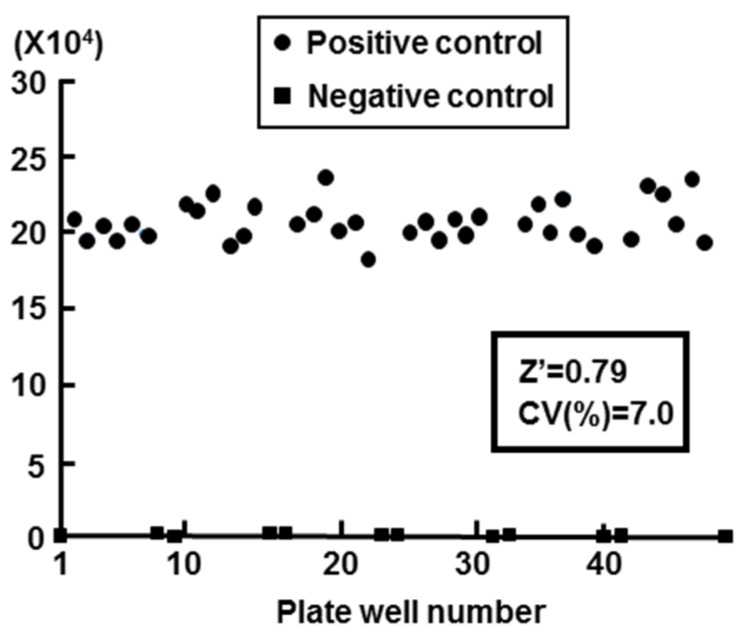
Validation of cell based HBV core promoter reporter assay. Huh7 GL4.18 CURS_BC_AeUS cells (positive control) and Huh7 GL4.18 cells (negative control) were harvested at 72 h. The luciferase activity was determined as described in the Experimental Section. The Z′ factor and coefficient of variation (CV) value was calculated as described in the Experimental Section.

We calculated the Z′ factor in order to evaluate the Huh7 G4.18 CURS_BC_AeUS cell line for high-throughput screening. The Z′ factor is a useful tool for measurement of the quality or suitability of high throughput screening, and the value spanning from 0.5 to 1.0 exhibits an appropriate assay [24,25]. In this study, the value of Z′ factor was 0.79 (*n* = 48) using Huh7 GL4.18 CURS_BC_AeUS cells (Figure 2). The coefficient of variation (CV), which represents unevenness of the screening system, should be less than 10% for a correct assay [24]. The CV value of our system was 7.0% (Figure 2).

HepG2.2.15 cell line is generally used to screen for anti-HBV agents, although HepG2.2.15 cell-based drug screening assay requires at least 10 days for screening. However, cell-based HBV core promoter assay was completed for 3 days for screening. The cell-based HBV core promoter assay is more advantageous than the assay using HepG2.2.15 cell in high-throughput screening of anti HBV agents. Thus, the cell-based HBV core promoter assay was employed in this study for high-throughput screening of extracts prepared from marine organisms.

### 2.3. High-Throughput Screening for Extracts of Marine Organisms Inhibiting HBV Core Promoter Activity

**Figure 3:**
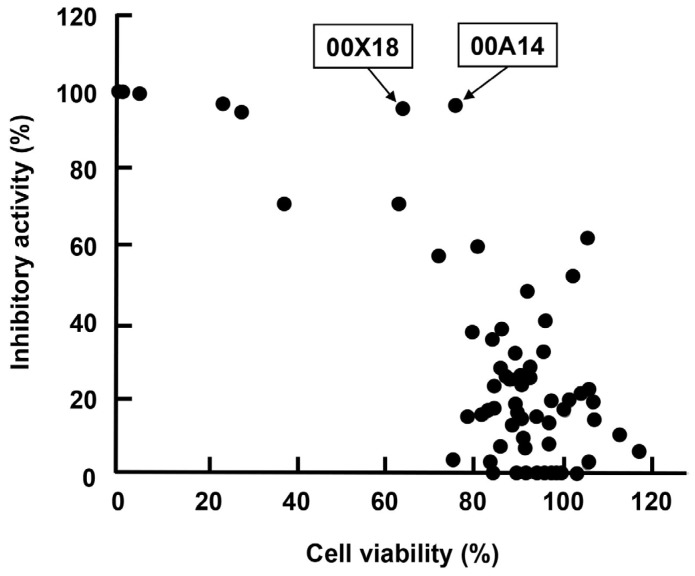
Correlation between the inhibitory activity of each marine organism extract against HBV core promoter and the cell viability of each marine organism extract. Each closed circle represents one marine organism extract. The *x*-axis indicates inhibitory activity against HBV core promoter, while the *y*-axis indicates cell viability.

We collected marine organisms from coral reefs of Indonesia and prepared 80 extracts from them with methanol (MeOH). We then screened them in order to discover anti-HBV agents using our screening system. Each extract was added at a final concentration of 25 µg/mL to the culture supernatant of Huh7 GL4.18 CURS_BC_AeUS cells. Luciferase activity and cell viability were measured 48 h after treatment. Among them, extracts of samples code named 00A14 and 00X18 exhibited high inhibitory activity of more than 95% and low cytotoxicity of 66% to 77% (Table 1, Figure 3). The 00A14 extract was prepared from the marine sponge *Dysidea granulosa* collected from the coral reefs of Simua Island, while the 00X18 extract was prepared from the marine sponge *Dysidea* sp. collected from the coral reefs of Buton strait. *Dysidea granulosa* of 00A14 was similar to *Dysidea* sp. of 00X18 regarding morphological features. The 00X18 extract, but not the 00A14 extract, was further analyzed in this study because of the much smaller amount of *Dysidea granulosa* than of *Dysidea* sp.

**Table 1:** Effect of marine organism extracts on HBV core promoter activity and cell viability.

Sample No.	Sample Code Name	Specimen	Phylum	Inhibitory Activity (%)	Cell Viability (% of Control)	Collection Site
1	00A01	*Callyspongia* sp.	Porifera	0	101.5	Simua Island
2	00A05	*Xestospongia* sp.	Porifera	2.3	97.7	Simua Island
3	00A07	*Ircinia ramosa*	Porifera	0	177.4	Simua Island
4	00A08	*Liosina* sp.	Porifera	99.4	0	Simua Island
5	00A09	*Clathria* sp*.*	Porifera	98.6	0	Simua Island
6	00A10	Unidentified	Chordata	18.1	108.3	Simua Island
7	00A11	*Hippospongia* sp*.*	Porifera	40.3	97.7	Simua Island
8	00A12	*Petrosia* sp.	Porifera	6.9	92.8	Simua Island
9	00A13	*Callyspongia cf.* *aerizusa*	Porifera	5.4	119.1	Simua Island
10	00A14	*Dysidea granulosa*	Porifera	96.5	77.3	Simua Island
11	00B15	*Spheciospongia vagabunda*	Porifera	0	104.8	Kajuongia Island
12	00B16	*Callyspongia* sp.	Porifera	0	101.3	Kajuongia Island
13	00B17	Unidentified	Porifera	0	91.2	Kajuongia Island
14	00J85	Unidentified	Porifera	0	96.6	Buton Island
15	00J86	Unidentified	Porifera	0	97.6	Buton Island
16	00J87	Unidentified	Porifera	0	95.2	Buton Island
17	00J88	*Phyllospongia* sp.	Porifera	17.9	90.7	Buton Island
18	00J89	Unidentified	Porifera	0	101.3	Buton Island
19	00J90	Unidentified	Porifera	0	96.3	Buton Island
20	00J91	*Parazoanthus* sp.	Cnidaria	24.2	90.1	Buton Island
21	00K92	Unidentified	Porifera	14.8	95.9	Buton Island
22	00K94	*Ianthella basta*	Porifera	94.2	28.8	Tobea Island
23	00K95	Unidentified	Chordata	9.2	92.4	Tobea Island
24	00K97	*Higginsia mixta*	Porifera	16.1	84.5	Tobea Island
25	00L00	*Thrinacophora cervicornis*	Porifera	0	85.7	Magintin Island
26	00L02	Unidentified	Chordata	3.1	76.6	Magintin Island
27	00M03	*Gelliodes fibulata*	Porifera	13.7	98.2	Masaloka Island
28	00M04	*Clavularia viridis*	Cnidaria	99.6	1.4	Masaloka Island
29	00M05	*Coelocarteria* sp.	Porifera	15.1	91.1	Masaloka Island
30	00M06	*Mycale* sp.	Porifera	37.6	87.6	Masaloka Island
31	00M07	Unidentified	Porifera	28.2	94.2	Masaloka Island
32	00M08	Unidentified	Porifera	96.3	24.3	Masaloka Island
33	00N09	Unidentified	Porifera	15.3	83.7	Buton strait
34	00N10	*Myrmekioderma granulatum*	Porifera	0	92.9	Buton strait
35	00N11	*Callyspongia samarensis*	Porifera	0	95.5	Buton strait
36	00N12	*Biemna* sp.	Porifera	6.8	87.5	Buton strait
37	00N13	*Biemna triraphis*	Porifera	24.1	92.6	Buton strait
38	00N14	*Xestospongia exigua*	Porifera	99.4	1.6	Buton strait
39	00P16	Unidentified	Cnidaria	0	101.0	Muna Island
40	00Q17	Unidentified	Porifera	16.1	101.9	Buton strait
41	00Q18	Unidentified	Porifera	2.5	84.8	Buton strait
42	00Q19	*Axinyssa* sp.	Porifera	51.5	104.0	Buton strait
43	00Q20	*Mycale* sp.	Porifera	22.7	86.0	Buton strait
44	00R22	*Clavularia inflate*	Cnidaria	22.1	107.0	Buton Island
45	00R23	*Paralemnalia* sp.	Cnidaria	19.2	99.0	Buton Island
46	00R24	*Junceella fragilis*	Cnidaria	18.6	102.9	Buton Island
47	00R25	*Nephthea* sp.	Cnidaria	0	94.6	Buton Island
48	00S26	*Svenzea* sp.	Porifera	25.5	88.5	Tobea Island
49	00S27	Unidentified	Cnidaria	32.0	97.3	Tobea Island
50	00S28	*Coelogorgia* sp.	Cnidaria	0	97.7	Tobea Island
51	00T29	*Theonella* sp.	Porifera	35.3	85.4	Tobea Island
52	00T30	Unidentified	Porifera	47.6	93.6	Tobea Island
53	00T31	*Higginsia cf.* *mixta*	Porifera	14.7	142.0	Tobea Island
54	00T32	*Paratelesto* sp.	Cnidaria	0	94.7	Tobea Island
55	00U33	*Pycnoclabella* sp.	Chordata	25.8	91.9	Muna Island
56	00U34	*Lissoclinum patella*	Chordata	27.8	87.2	Muna Island
57	00X01	*Polycarpa contecta*	Chordata	25.9	93.6	Simua Island
58	00X02	*Dysidea* sp.	Porifera	56.8	73.4	Tobea Island
59	00X04	*Nephthea* sp.	Cnidaria	0	99.2	Beromasidi Island
60	00X05	*Haliclona fascigera*	Porifera	7.8	98.7	Torobulu
61	00X06	*Axinyssa* sp.	Porifera	98.9	4.9	Torobulu
62	00X07	Unidentified	Porifera	16.7	86.0	Torobulu
63	00X08	Unidentified	Cnidaria	12.9	87.0	Torobulu
64	00X10	*Niphates olemda*	Porifera	70.2	38.5	Buton Island
65	00X11	Unidentified	Porifera	99.7	1.1	Tobea Island
66	00X12	Unidentified	Porifera	70.6	63.9	Tobea Island
67	00X13	Unidentified	Porifera	13.9	108.7	Magintin Island
68	00X14	*Xestospongia* sp.	Porifera	21.1	105.6	Magintin Island
69	00X15	*Dysidea*/*Euryspongia*	Porifera	10.2	114.4	Magintin Island
70	00X16	Unidentified	Chordata	0	130.3	Buton strait
71	00X17	*Dysidea* *cf.* *arenaria*	Porifera	14.7	80.0	Buton strait
72	00X18	*Dysidea* sp.	Porifera	95.0	65.3	Buton strait
73	00X19	Unidentified	Porifera	23.0	92.5	Buton strait
74	00X21	*Gelliodes/Niphates*	Porifera	36.7	80.9	Buton strait
75	00X22	*Amphimedon/Haliclona*	Porifera	31.2	90.9	Buton strait
76	00X23	*Dysidea cf. arenaria*	Porifera	0	92.8	Buton strait
77	00X24	Unidentified	Porifera	0	99.2	Buton strait
78	00X26	*Anthelia* sp.	Cnidaria	61.5	107.4	Buton strait
79	00X27	Unidentified	Chordata	14.8	85.2	Tobea Island
80	00X28	*Clathria* sp.	Porifera	58.9	82.0	Tobea Island

### 2.4. Identification of PBDEs as the Inhibitory Compounds of HBV Production via HBV Core Promoter Activity

**Figure 4:**
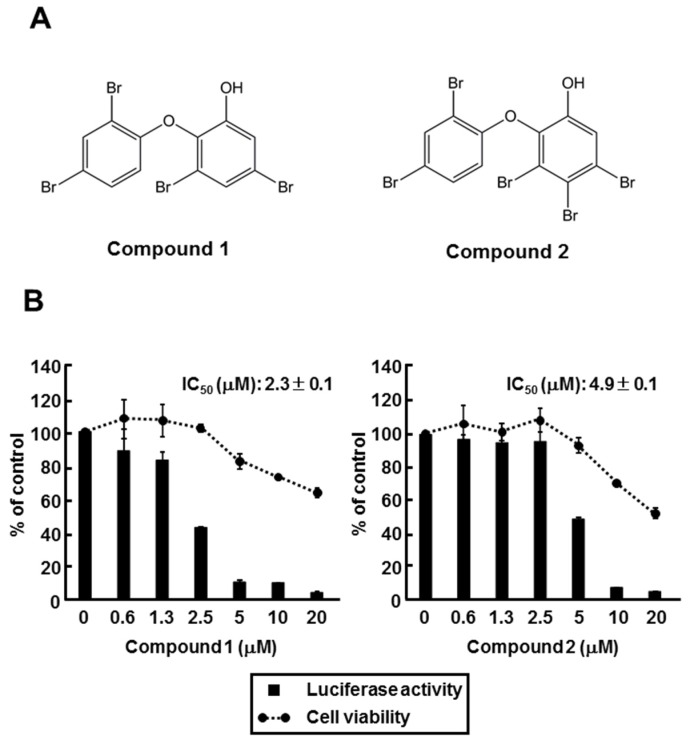
Effect of polybrominated diphenyl ethers (PBDEs) on HBV core promoter activity. (**A**) Structure of 3,5-dibromo-2-(2,4-dibromo-phenoxy)-phenol (Compound **1**) and 3,4,5-tribromo-2-(2,4-dibromo-phenoxy)-phenol (Compound **2**); (**B**) Huh7 GL4.18 CURS_BC_AeUS cells were incubated for 48 h in the medium containing various concentrations of PBDEs. Luciferase and cytotoxicity assays were carried out by the method described in the Experimental section. Data are representative of three independent experiments. Error bars indicate standard deviation.

The extract of 00X18 was separated with several chromatographic steps to give two polybrominated diphenyl ethers (PBDEs), 3,5-dibromo-2-(2,4-dibromophenoxy)-phenol (compound **1**) and 3,4,5-tribromo-2-(2,4-dibromophenoxy)-phenol (compound **2**) as major constituents (Figure 4A). The compounds were identified by comparing the NMR data with those published. Huh7 GL4.18 CURS_BC_AeUS cells were incubated with each of those compounds to evaluate their effects on the core promoter activity. Both compounds inhibited the core promoter activity in a dose-dependent manner (Figure 4B). IC_50_ values of compounds **1** and **2** are 2.3 µM and 4.9 µM, respectively, suggesting that compounds **1** and **2** included in the 00X18 extract inhibit HBV core promoter activity.

**Figure 5:**
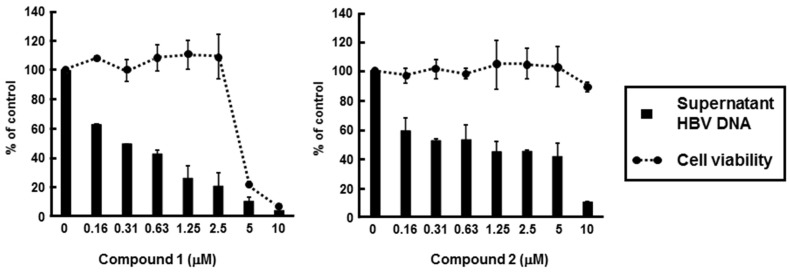
Effect of PBDEs on HBV production. HepG2.2.15.7 cells were incubated with various concentrations of compound **1** or **2**. Supernatant HBV DNA and cytotoxicity were estimated by real-time qPCR and MTS assay, respectively, as described in the Experimental section. The data were representative of three independent experiments. Error bars indicate standard deviation.

We next addressed the effects of compounds **1** and **2** on HBV production and cell viability. HBV-producing cultured cells, HepG2.2.15.7, were incubated in culture medium containing various concentrations of compound **1** or **2**. Entecavir was used as the positive control for anti-HBV activities of compound **1** and **2**. The amount of supernatant HBV DNA and cell viability were measured by using real-time PCR and MTS assay, respectively. Treatment with compound **1** or **2** impaired production of HBV DNA in a dose-dependent manner (Figure 5). The IC_50_ and CC_50_ values of compound **1** were 0.23 µM and 4.19 µM, respectively, while the EC_50_ and CC_50_ values of compound **2** were 0.80 µM and 10.26 µM, respectively. Thus, the selectivity indexes of compounds **1** and **2** were 18.2 and 12.8, respectively (Table 2). These results suggest that compounds **1** and **2** possess anti-HBV activity. However, IC_50_ values of compound **1** and **2** were higher than that of entecavir, while compounds **1** and **2** were more toxic than entecavir (Table 2).

**Table 2:** Anti HBV activity and cytotoxicity of Compound **1**, **2** and entecavir in HepG2.2.15.7 cells.

Compound	EC_50_^a^ (µM)	CC_50_^b^ (µM)	Selectivity ^c^ Index
Compound **1**	0.23 ± 0.07	4.19 ± 0.12	18.2
Compound **2**	0.80 ± 0.34	10.26 ± 3.69	12.8
Entecavir	0.021 ± 0.003	>100	>4761

^a^ Fifty percent effective concentration based on the inhibition of the HBV viral DNA release; ^b^ Fifty percent cytotoxicity concentration based on the reduction of cell viability; ^c^ Selectivity index (CC_50_/EC_50_).

HepG2.2.15 cells have generally been used to screen chemical compounds for anti-HBV agents, but the disadvantage of HepG2.2.15 cell-based drug screening assay requires at least 10 days for screening. However, cell-based HBV core promoter assay was completed for 2 days for screening. Thus, cell-based HBV core promoter assay offers an advantage in high-throughput screening of anti HBV agents.

PBDEs were recently isolated from marine sponges and biologically synthesized by their associated microorganisms [26,27]. Several groups reported multifunctional properties containing antibacterial, antifungal, anti-microalgal and anti-inflammatory activities of PBDEs [28,29,30]. Treatment with PBDEs also inhibited the enzymatic activities of endogenous and viral proteins [31,32,33]. Compound 1 suppressed activity of Tie2 kinase, which is associated with angiogenesis essential for tumor growth and survival [34]. The data reported by Zhang *et al.*, indicate that compound 1 induces G1 phase cell cycle arrest in MCF-7 cells (a breast cancer cell line) [35], although HBV could infect and replicate in non-dividing cells [36]. These reports indicate that endogenous factors are associated with the inhibitory effect of PBDEs on HBV propagation. Further studies will reveal the mechanism of PBDE-related suppression of HBV production, and will be required for the development of more effective and safe anti-HBV agents based on PBDEs.

## 3. Experimental Section

### 3.1. Cell Culture

HepG2.2.15.7 cell line was subcloned from HepG2.2.15 cell line, which is stably transfected with the HBV genome (genotype D) [37,38]. HepG2.2.15.7 cell line produced HBV at a higher level than HepG2.2.15 cell line. This cell lines were maintained in Dulbecco’s Modified Eagle’s Medium/Ham’s Nutrient Mixture F12 medium supplemented with 10% fetal bovine serum, 100 U/mL Penicillin, 100 µg/mL Streptomycin, 2 mM l-Glutamine, 400 µg/mL G418, 50 µM hydrocortisone and 5 µg/mL Insulin. Huh7 cells, HeLa cells and HT-1080 cells were maintained in Dulbecco’s modified Eagle’s medium containing 10% fetal calf serum, 100 U/mL Penicillin and 100 µg/mL Streptomycin.

### 3.2. Plasmid Construction and Transient or Stable Expression

The HBV CURS BCP and BCP fragment was amplified from pUC19 HBV AeUS plasmid [39] by PCR using the following primers: CURS BCP: 5′-GCTAGCGATCCTGCCCAAGGTCTTACATAA-3′ (the underlined region indicates Nhe I site) and 5′-AGATCTAAGAGATGATTAGGCAGAGGT-GAA-3′ (underlined region indicates Bgl II site); BCP: 5′-GCTAGCTGGGGGAGGAGATTAGGT-TAAAGG-3′ (the underlined region indicates Nhe I site) and 5′-AGATCTAAGAGATGATTAGGC-AGAGGTGAA-3′ (underlined region indicates Bgl II site). These PCR products were cloned into a TA cloning vector, pTA2 (TOYOBO, Osaka, Japan). After sequence confirmation, these PCR fragments were introduced between Nhe I and Bgl II sites of pGL4.18 [*luc2P*/Neo] (Promega, Madison, WI, USA). The resulting plasmid was designated as pGL4.18 CURS_BC_AeUS and pGL4.18 BC_AeUS in this study.

Huh7, HeLa and HT-1080 cell lines were co-transfected with pGL4.18 CURS_BC_AeUS and phRG-TK using Lipofectamine LTX reagent (Thermo Fisher Scientific, Waltham, MA, USA). To standardize transfection efficiency and cell recovery, we used the phRG-TK plasmid (Promega, Madison, WI, USA) encoding *Renilla* luciferase under the control of the herpes simplex virus type 1 thymidine kinase promoter. The plasmid pGL4.18 [*luc2P*/Neo] was used as a negative control instead of pGL4.18 CURS_BC_AeUS. The transfected cells were harvested 48 h post-transfection and then were lysed in Passive lysis buffer (Promega, Madison, WI, USA). Luciferase activity was measured using a Dual-luciferase reporter assay system (Promega, Madison, WI, USA). The resulting luminescence was detected by a Luminescencer-JNR AB-2100 (ATTO, Tokyo, Japan).

The pGL4.18 CURS_BC_AeUS or pGL4.18 [*luc2P*/Neo] plasmid was transfected into Huh7 cells using Lipofectamine LTX reagent (Thermo Fisher Scientific, Waltham, MA, USA). These transfected cells were seeded on the plate and then incubated until colonies formed. The stable cell lines were established by colony isolation. The clone exhibiting the highest activity among the isolated clone was designated as Huh7 GL4.18 CURS_BC_AeUS, and the negative control clone was designated as Huh7 GL4.18.

### 3.3. Validation of Screening Method

Huh7 GL4.18 CURS_BC_AeUS and Huh7 cells were seeded at 2 × 10^4^ cells per well in a 48-well plate. Luciferase activity was measured after 72 h of incubation. The Z′ factor was calculated as follows:

$$Z^{\prime }\;=\;\text{1 − }\frac{\left(3\times SD{\;}_{luciferase\;activity\;of\;Huh7\;GL4.18\;CURS\_BC\_AeUS})\;+\;(3\times SD{\;}_{luciferase\;activity\;of\;Huh7\;GL4.18}\right)}{\left(mean{\;}_{luciferase\;activity\;of\;Huh7\;GL4.18\;CURS\_BC\_AeUS})\;-\;(mean{\;}_{luciferase\;activity\;of\;Huh7\;GL4.18}\right)}$$

SD: Standard Deviation.

The minimal acceptable value for a high-throughput screening assay is usually considered to be 0.5. The theoretical maximum is 1 [24,25].

The CV is calculated using the formula:

$$CV\;\left(\%\right)\;=\;\frac{SD{\;}_{luciferase\;activity\;of\;Huh7\;GL4.18\;CURS\_BC\_AeUS}}{mean{\;}_{luciferase\;activity\;of\;Huh7\;GL4.18\;CURS\_BC\_AeUS}}\;\times \;100$$

SD: Standard Deviation.

The acceptable value of CV for a high-throughput screening assay is less than 10% [24].

### 3.4. Cell-Based HBV Promoter Assay

Huh7 GL4.18 CURS_BC_AeUS cells were seeded at 2 × 10^4^ cells per well in a 48-well plate and then treated with 25 µg/mL each extracts 24 h after seeding cells. The treated cells were harvested 48 h post-treatment and then lysed with Cell culture lysis buffer (Promega, Madison, WI, USA). Luciferase activity was measured by using Luciferase assay systems (Promega, Madison, WI, USA). The resulting luminescence was detected as described above.

### 3.5. Determination of Cytotoxicity

Huh7 GL4.18 CURS_BC_AeUS cells were seeded at a density of 1 × 10^4^ cells per well in a 96-well plate and incubated at 37 °C for 24 h. Each extract was added at 25 µg/mL to the culture supernatant. The treated cells were harvested 48 h post-treatment. Cell viability was estimated by dimethylthiazol carboxymethoxy-phenylsulfophenyl tetrazolium (MTS) assay using a Celltiter 96 aqueous one-solution cell proliferation assay kit (Promega, Madison, WI, USA).

### 3.6. Preparation of Extracts from Marine Organisms

The marine organisms were collected at coral reefs around Sulawesi, Muna, and Buton Islands, Indonesia, in August 2000. Marine sponge No. 10 was identified as Dysidea granulosa in this study and deposited at the Netherlands Centre for Biodiversity with code RMNH POR 10013. Each specimen was preserved with a small amount of ethanol until use. After decantation of ethanol solution, each specimen was extracted three times with MeOH. A crude extract was prepared by concentrating the combined solution under vacuum and then kept at −20 °C until use. A portion of each extract was solubilized in dimethyl sulfoxide (DMSO) after measuring its weight.

### 3.7. Separation of PBDEs

A crude MeOH extract (1.24 g) of specimen No. 72 was partitioned between EtOAc and water. The lipophilic layer yielded 481 mg after concentration and it was applied to a silica gel column and separated into six fractions. A portion of the third fraction (185 mg) was subjected to silica HPLC (hexane-CH2Cl2 or hexane-EtOAc). Finally, two PBDEs, 3,5-dibromo-2-(2,4-dibromo-phenoxy)-phenol (Compound **1**, 2.5 mg) and 3,4,5-tribromo-2-(2,4-dibromo-phenoxy)-phenol (Compound **2**, 7.3 mg), were identified by checking NMR data as follows:

Compound **1**: ^1^H NMR (acetone-*d*_6_) δ 7.83 (^1^H, d, *J* = 2.4 Hz, H-3′), 7.43 (^1^H, dd, *J* = 8.8, 2.4 Hz, H-5′), 7.39 (^1^H, d, *J* = 2.2 Hz, H-4), 7.26 (^1^H, d, *J* = 2.2 Hz, H-6), 6.59 (^1^H, d, *J* = 8.8 Hz, H-6′).

Compound **2**: ^1^H NMR (acetone-*d*_6_) δ 7.83 (^1^H, d, *J* = 2.4 Hz, H-3′), 7.51 (^1^H, s, H-6), 7.42 (^1^H, dd, *J* = 8.8, 2.4 Hz, H-5′), 6.63 (^1^H, d, *J* = 8.8 Hz, H-6′).

### 3.8. Determination of Anti HBV Activity and Cytotoxicity in HepG2.2.15.7 Cells

HepG2.2.15.7 cells were seeded at 1 × 10^4^ cells per well in a collagen coated 96-well plate (Corning, Corning, NY, USA) and incubated at 37 °C for 24 h before treatment. The tested compound was added to the culture medium at the indicated concentrations. The culture medium was exchanged every 3 days for fresh medium containing the compound. The treated cells and culture supernatants were harvested 9 days post-treatment and were subjected to MTS assay and estimation of HBV DNA, respectively. The culture supernatant was mixed with an equal volume of Sidestep lysis and stabilization buffer (Agilent Technologies, Santa Clara, CA, USA). The viral DNA included in the mixture was estimated by real-time quantitative PCR using the THUNDERBIRD Probe qPCR Mix (TOYOBO, Osaka, Japan). The forward and reverse primers targeting HBV surface region are 5′-ACTCACCAACCTCCTGTCCT-3′ and 5′-GACAAACGGGCAACATACCT-3′, respectively. The fluorogenic probe was 5′-FAM-TATCGCTGGATGTGTCTGCGGCGT-TAMRA-3 [40].

### 3.9. Reagents

Entecavir and hydrocortisone were purchased from Sigma-Aldrich (St. Louis, MO, USA). G418 and insulin were purchased from Wako (Osaka, Japan).

## 4. Conclusions

We developed a cell-based assay based on HBV core promoter activity, screened marine products using this assay system and finally identified two PBDEs as anti-HBV compounds.
